# The Anatomy of the Arteries of the Human Body, and Its Applications to Pathology and Operative Surgery; with a Series of Lithographic Drawings the Size of Nature

**Published:** 1846-10

**Authors:** 


Art. V.—*The Anatomy of the Arteries of the Human Body, and its Applications to Pathology and Operative Surgery; with a series of Lithographic Drawings the Size of Nature.* By Richard Quain, f.r.s., The Drawings by Joseph Maclise, Esq., Surgeon. Eighty-seven Plates, imperial folio. The Letter-press 8vo, pp. 550. *London,* 1844.

Although we have already given a full and elaborate analysis of this incomparable work (No. XXXVIII, April, 1845), we are induced to notice it once more, from the circumstance of its now coming before us, virtually, though not actually, as a New Edition, and because we feel it a duty to our readers, to make them acquainted with the splendid prize now within their reach. The publishers, in their new Prospectus, inform us "that as the large size of the stones on which the drawings are made, renders it difficult to retain them uninjured for use from time to time, whenever they may be wanted, they propose to throw off at once 500 copies, and then efface the drawings from the stones, that the plates may not be produced in a less perfect manner." When subscribers are received for these copies (500), the work will be delivered at the price of Six Guineas, in place of the original price of £10 12s. This, we will venture to say, from our own knowledge, is one of the cheapest publications ever offered to the profession; and we earnestly advise *every surgeon who operates or expects to operate,* not to lose the present opportunity of possessing a work which may be really said to be almost indispensable. The *possibility* of obtaining it can only exist for a short time, as there cannot be a doubt but the small subscription list of five hundred will soon be filled. We would suggest to the publishers to offer the work even at a less price, say *five guineas,* provided the subscribers (as seems likely) greatly exceed the stipulated number of five hundred.

